# Tobacco exposure and alcohol drinking prevalence and associations with hypertension in rural southwest China: A cross-sectional study

**DOI:** 10.18332/tid/189222

**Published:** 2024-06-10

**Authors:** Guohui Li, Lan Liu, Du-li Liu, Zi-zi Yu, Allison R. Golden, Xiang-yang Yin, Le Cai

**Affiliations:** 1 NHC Key Laboratory of Drug Addiction Medicine, Kunming Medical University, Kunming, Yunnan, China; 2 Yunnan Provincial Key Laboratory of Public Health and Biosafety, School of Public Health, Kunming Medical University

**Keywords:** smoking, exposure to secondhand smoke, alcohol drinking, hypertension, China

## Abstract

**INTRODUCTION:**

This study examined the prevalence of tobacco exposure and drinking and ascertained the relationships between tobacco exposure, alcohol drinking, concurrent smoking and drinking, and hypertension in rural southwestern China.

**METHODS:**

Data were collected from a cross-sectional health interview and examination survey, which included 7572 adults aged ≥35 years, in rural China. Participant demographic characteristics, smoking habits, exposure to secondhand smoke (SHS), and alcohol drinking habits were obtained using a standard questionnaire. Blood pressure (BP), height, weight, and waist circumference were measured for each participant.

**RESULTS:**

The overall prevalence of smoking, SHS exposure, drinking, concurrent smoking and drinking, concurrent exposure to SHS and drinking, and hypertension was 37.7%, 27.4%, 16.2%, 12.6%, 1.6%, and 41.3%, respectively. Males had a significantly higher prevalence of smoking (74.1% vs 2.2%, p<0.01), drinking (31.1% vs 1.7%, p<0.01), and concurrent smoking and drinking than females (25.3% vs 0.3%, p<0.01). However, females had a higher prevalence of SHS exposure than males (30.2% vs 20.6%, p<0.01). Ethnic minorities had a higher prevalence of SHS exposure, drinking, and concurrent smoking and drinking, than Han participants (p<0.01). Participants with a higher education level had a higher prevalence of smoking, drinking, and concurrent smoking and drinking than their counterparts (p<0.01). In contrast, participants with a lower education level had a higher prevalence of SHS exposure than their counterparts (p<0.01). Multivariate logistic regression analysis found that smokers (AOR=1.31; 95% CI: 1.13–1.51), individuals exposed to SHS (AOR=1.24; 95% CI: 1.11–1.43), drinkers (AOR=1.31; 95%: CI: 1.15–1.50), and concurrent smokers and drinkers (AOR=1.45; 95% CI: 1.25–1.67) all had a higher probability of having hypertension (p<0.01). Additionally, concurrent smoking and drinking had the strongest association with the prevalence of hypertension (AOR=1.45; 95% CI: 1.25–1.67; p<0.01).

**CONCLUSIONS:**

Socioeconomic factors play an important role in influencing the prevalence of smoking, exposure to SHS, and drinking in rural southwest China. Interventions to prevent and reduce hypertension should, in particular, focus on smokers, individuals exposed to SHS, drinkers, and, in particular, concurrent smokers and drinkers.

## INTRODUCTION

Tobacco use is a major public health challenge worldwide. Smoking and exposure to secondhand smoke (SHS) both increase the risk of disease, including cancer, cardiovascular diseases, and chronic respiratory diseases^1^. Globally, there were 1.18 billion frequent smokers in 2020, which contributed to 7 million fatalities and roughly one-seventh of all deaths that year^2^. With China having more than one-third of the global tobacco consumption in 2019, the country is both the world’s largest producer and consumer of tobacco^3^. According to China’s 2018 national survey, 68.1% of Chinese non-smokers also reported regular exposure to SHS^4^. China’s rural areas are the hardest hit by tobacco; rural areas consistently had a higher smoking prevalence than metropolitan areas from 2007 to 2018^5^. In the absence of widespread smoking cessation, China’s tobacco-related mortality is expected to rise from about 1 million in 2010 to 3 million by 2050^6^.

Excessive alcohol drinking is also a serious global public health issue. Alcohol drinking was the seventh leading risk factor for death and disability worldwide^7^. About 3 million alcohol-related deaths occurred globally in 2016, and the number of disability-adjusted life years exceeded 130 million^8^. Many low- and middle-income countries have seen increases in alcohol consumption in the last 20 years, with men the major consumers^9^. The prevalence of alcohol drinking among Chinese adults has risen in recent years, reaching 43.7% from 2015 to 2017, an increase of 13.2% compared to 2010 to 2012^10^. Moreover, urban areas have a higher alcohol drinking prevalence than rural areas (46.5% vs 40.8%), and men have a higher drinking prevalence than females (64.5% vs 23.1%)^11^. Previous research has linked alcohol consumption to an increased risk of 61 diseases among Chinese men, including cardiovascular, respiratory, and digestive diseases^12^.

China has the highest number of hypertensive patients in the world, with a prevalence rate of 32% for females and 37% for males, exceeding the global average of 20% for females and 24% for males^13^. Smoking and alcohol drinking have both been recognized as risk factors for hypertension in previous studies^14^. Additionally, alcohol use is frequently higher among smokers^15^, and smoking and drinking concurrently greatly raises the risk of developing hypertension^16^. Exposure to SHS can also increase the risk of hypertension, especially among women, young people, and the elderly^17^.

Yunnan Province is the largest tobacco producer in China and a pillar industry in the region. The impact of tobacco on health is also among the gravest in Yunnan. Yunnan Province has the largest ethnic minority population in China. Previous research had found the prevalence of hypertension in Yunnan Province increased from 26.1% in 2011 to 40.4% in 2021^18^, and the prevalence of smoking and alcohol drinking in the region is higher than other provinces in China. The prevalence of SHS exposure in Yunnan Province was also higher than the average level in China (82.8% vs 68.1%). At present, there are limited data on the relationship between hypertension and tobacco exposure and alcohol drinking in rural southwestern China. Thus, this study examined the prevalence of tobacco exposure (including smoking and SHS exposure) and alcohol drinking, and simultaneously investigated the relationship of tobacco exposure, alcohol drinking, and concurrent smoking and drinking with hypertension among southwest China’s rural adult population aged ≥35 years.

## METHODS

### Study area and population

This study was conducted in three counties of Yunnan Province from 2020 to 2021 using a cross-sectional health interview and examination survey. A multi-stage stratified random sampling technique was used to select for participation rural residents aged ≥35 years from each selected village’s committee. In the first stage, Yunnan Province rural population was divided into three categories according to per capita gross domestic product (GDP): high, medium, and low. One county was then randomly selected from each of these categories for a total of three selected counties. In the second stage, one township was randomly selected from each of these three categories, for a total of nine. In the third stage, three villages were chosen by probability proportional to size (PPS) from each of the nine townships. In the fourth stage, simple random sampling was conducted to select the sample subjects from a village based on a list of individuals aged ≥35 years obtained from each selected village’s committee. This sampling method has been detailed previously^19^.

### Sample size calculation

The formula for a cross-sectional study was used to calculate the sample size for each selected village:


$$n=\frac{Z_{1-\alpha /2}^{2}\times p(1\text{-}p)}{\delta^{2}}\times deff$$

where p is the prevalence of hypertension in the Chinese population^13^, *δ* is the margin of error (to estimate prevalence with a precision is equal to half of the prevalence of hypertension), and *deff* is the effect of design (*deff*=2).

### Data collection and measurement

A pre-tested and structured questionnaire was used in face-to-face interviews conducted by trained interviewers with participants who provided informed consent. It showed satisfactory psychometric properties, with strong internal consistency through a Cronbach’s alpha of 0.83, and had high test-retest reliability, as evidenced by a high correlation coefficient of 0.92 for the total scale. Age, sex, ethnicity, annual household income, education level, family history of hypertension, self-reported smoking and alcohol drinking behaviors, and exposure to SHS were all included in the questionnaire. Height, weight, and blood pressure (BP) measurements were obtained for anthropometry. All measurements were carried out in accordance with international standardized procedures. Height and weight were measured using standardized methods and following the WHO STEPS^20^.

The American Heart Association’s suggested technique of measuring BP three times consecutively was used and the average of the three measurements was recorded to calculate the participants’ BP^21^.

### Definitions

Hypertension was defined as average systolic blood pressure (SBP) of ≥140 mmHg, and/or diastolic blood pressure (DBP) of ≥90 mmHg, and/or currently receiving antihypertensive treatment, in accordance with the Global Guidelines for Hypertension Practice. A former diagnosis of hypertension at a qualified medical institution was also defined as hypertension in our study. Smoking was defined as having smoked continuously or cumulatively for six months or more and smoking more than 100 cigarettes in total, following WHO standards. Current smokers were defined as participants who smoked any type of tobacco product daily during the survey period. SHS exposure was defined as a report of exposure to ambient tobacco smoke for at least 15 minutes, one day a week, at work or home, by a non-smoker. Current alcohol drinkers were defined as those who consumed alcohol for 12 days or more in the year preceding the survey. Body mass index (BMI) was calculated as weight (kg) divided by height squared (m^2^); participants with a BMI ≥28 kg/m^2^ were defined as obese. Illiteracy is the inability to read with understanding or write simple sentences about everyday life. Annual household income was defined as either low or high, with the median value as the cut-off point.

### Statistical analysis

EpiData 3.1 software was used for double data entry, and SPSS 22.0 software was used for data analysis. Mean values of SBP, DBP, and BMI were expressed as mean ± standard deviation. Categorical variables were described as frequencies and percentages. Chi-squared tests were used to compare categorical variables while independent samples Student’s t-tests were used to analyze continuous measures between two groups. Multivariate logistic regression was used to analyze the association of smokers, exposure to SHS, drinkers, and concurrent smoking and drinking with the prevalence of hypertension, adjusted for age, sex, ethnicity, education level, level of annual household income, family history of hypertension, and obesity. The factors associated with hypertension were based on biological plausibility and initially assessed using univariable logistic regression analysis. Variables with p<0.05 in the univariable analysis were considered candidates for the multivariate logistic regression. Interactions were tested to determine whether the associations between smoking or drinking and the prevalence of hypertension differ by sex. Associations were expressed as adjusted odds ratios (AOR) and 95% confidence intervals (CI). All statistical significance decisions were based on a two-tailed p<0.05.

## RESULTS

A total of 7704 individuals aged ≥35 years were selected via the sampling process. Of these, 7572 people consented to participate, with a response rate of 98.29%.

Table 1 summarizes the demographic characteristics of the participants. A total of 3739 males (49.4%) and 3833 females (50.6%) participated. Han participants accounted for 54.5%, while ethnic minorities accounted for 44.5%. Males had a higher level of education than females, whereas females had a higher prevalence of family history of hypertension, prevalence of obesity, and mean BMI than males (p<0.01).

**Table 1:** General characteristics, mean blood pressure, and anthropometric measurements in adults aged ≥35 years from a 2020–2021 cross-sectional study in rural Yunnan Province, China (N=7572)

*Characteristics*	*Male n (%)*	*Female n (%)*	*All n (%)*
**Total**	3739 (49.4)	3833 (50.6)	7572 (100)
**Age^a^** (years)			
35–44	569 (15.2)	687 (17.9)	1256 (16.6)
45–54	989 (26.5)	916 (23.9)	1905 (25.2)
55–64	936 (25.0)	920 (24.0)	1856 (24.5)
65–74	810 (21.7)	860 (22.4)	1670 (22.1)
≥75	435 (11.6)	450 (11.7)	885 (11.7)
**Ethnicity^a^**			
Han	2035 (54.4)	2089 (54.5)	4124 (54.5)
Minority	1704 (45.6)	1744 (45.5)	3448 (45.5)
**Education level^a^**			
Illiterate	667 (17.8)	1063 (27.7)**	1730 (22.8)
Primary (grade 1–6) or higher	3072 (82.2)	2770 (72.3)	5842 (77.2)
**Annual household income^a^**			
Low	1877 (50.2)	1967 (51.3)	3844 (50.8)
High	1862 (49.8)	1866 (48.7)	3728 (49.2)
**Family history of hypertension^a^**	558 (14.9)	683 (17.8)**	1241 (16.4)
**Obesity^a^**	403 (10.8)	504 (13.1)**	907 (12.0)
**BMI^b^** (kg/m^2^), mean ± SD	23.6 ± 3.5**	23.9 ± 3.7**	23.7 ± 3.6**
**SBP^b^** (mmHg), mean ± SD	129.8 ± 21.6**	126.5 ± 22.7**	128.1 ± 22.2**
**DBP^b^** (mmHg), mean ± SD	81.3 ± 13.4**	78.1 ± 13.2	79.7 ± 13.4**

BMI: body mass index. SBP: systolic blood pressure. DBP: diastolic blood pressure.

a Difference of age, ethnicity, education level, annual household income, family history of hypertension and obesity between two sexes were analyzed by chi-square test.

b Difference of BMI, SBP and DPB between the two sexes were analyzed by independent two-sample t-test.

Statistical significance at

* p<0.05,

** p<0.01.

Table 2 presents the prevalence of smoking, SHS exposure, and drinking among the study population. The overall prevalence of smoking, SHS exposure, drinking, concurrent smoking and drinking, and concurrent exposure to SHS and drinking was 37.7%, 27.4%, 16.2%, 12.6%, and 1.6%, respectively. Males had a significantly higher prevalence of smoking (74.1% vs 2.2%, p<0.01), drinking (31.1% vs 1.7%, p<0.01), and concurrent smoking and drinking than females (25.3% vs 0.3%, p<0.01). In contrast, females had a higher prevalence of SHS exposure than males (30.2% vs 20.6%, p<0.01). Participants aged 45–54 years had the highest prevalence of smoking and SHS exposure, while participants aged 55–64 years had the highest prevalence of drinking and concurrent smoking and drinking (p<0.01). Ethnic minorities had a higher prevalence of SHS exposure, drinking, and concurrent smoking and drinking than the Han participants (p<0.01). Participants with a higher level of education had a higher prevalence of smoking, drinking, and concurrent smoking and drinking than their counterparts (p<0.01). In contrast, participants with a lower level of education had a higher prevalence of SHS exposure than their counterparts (p<0.01).

**Table 2:** Prevalence of smoking, secondhand smoke (SHS) exposure, and drinking among adults aged ≥35 years from a 2020–2021 cross-sectional study in rural Yunnan Province, China (N=7572)

*Variable*	*Smokers n (%)*	*Exposure to SHS n (%)*	*Drinkers n (%)*	*Concurrent smoking and drinking n (%)*
**Total**	2855 (37.7)	1461 (27.4)	1226 (16.2)	957 (12.6)
**Sex**				
Male	2769 (74.1) **	318 (20.6)	1161 (31.1) **	947 (25.3) **
Female	86 (2.2)	1143 (30.2) **	65 (1.7)	10 (0.3)
**Age** (years)				
35–44	406 (32.3)	264 (29.5)	160 (12.7)	127 (10.1)
45–54	773 (40.6) **	378 (30.4) **	326 (17.1)	257 (13.5)
55–64	739 (39.8)	372 (29.5)	362 (19.5) **	294 (15.8) **
65–74	624 (37.4)	301 (24.2)	250 (15.0)	188 (11.3)
≥75	313 (35.4)	146 (21.2)	128 (14.5)	91 (10.3)
**Ethnicity**				
Han	1518 (36.8)	403 (22.8)	558 (13.5)	451 (10.9)
Minority	1337 (38.8)	1185 (33.1) **	668 (19.4) **	506 (14.7) **
**Education level**				
Illiterate	514 (29.7)	372 (28.1)	234 (13.5)	171 (9.9)
Primary (grade 1–6) or higher	2341 (40.1) **	1089 (27.2)	992 (17.0) **	781 (13.5) **
**Annual household income**				
Low	1458 (37.9)	777 (28.7) **	646 (16.8)	509 (13.2)
High	1397 (37.5)	684 (26.1)	580 (15.0)	448 (12.0)

Differences between smokers, exposure to SHS, drinkers and concurrent smoking and drinking were analyzed by chi-squared test.

Statistical significance at

* p<0.05,

** p<0.01.

**Figure 1:**
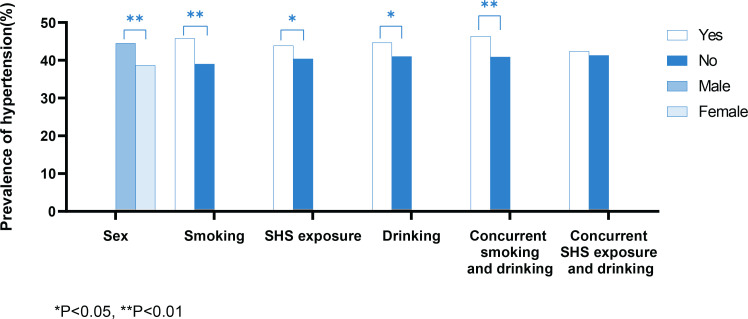
Prevalence of hypertension by sex, smoking, secondhand smoke exposure (SHS), and drinking status among adults aged ≥35 years from a 2020–2021 cross-sectional study in rural Yunnan Province, China (N=7572)

Table 3 and Figure 1 indicate the prevalence of hypertension by sex, smoking, SHS exposure, and alcohol drinking status. The overall prevalence of hypertension in the surveyed population was 41.6% (44.5% for males and 38.7% for females). Males had a higher prevalence of hypertension than females (p<0.01). Smokers, individuals exposed to SHS, drinkers, and concurrent smokers and drinkers had a higher prevalence of hypertension than their counterparts (p<0.05).

**Table 3:** Prevalence of hypertension by sex, smoking, secondhand smoke exposure (SHS), and drinking status among adults aged ≥35 years from a 2020–2021 cross-sectional study in rural Yunnan Province, China (N=7572)

*Variable*	*Hypertension n (%)*	*No Hypertension n (%)*
**Sex**		
Male	1663 (44.5) **	2076 (55.5)
Female	1485 (38.7)	2348 (61.3)
**Smoker**		
Yes	1308 (45.8) **	1547 (54.2)
No	1840 (39.0)	2877 (61.0)
**SHS exposure**		
Yes	641 (43.9) *	820 (56.1)
No	1565 (40.4)	2307 (59.6)
**Drinker**		
Yes	548 (44.7) *	678 (55.3)
No	2600 (41.0)	3746 (59.0)
**Concurrent smoking and drinking**		
Yes	444 (46.4) **	513 (53.6)
No	2704 (40.9)	3911 (59.1)
**Concurrent SHS exposure and drinking**		
Yes	36 (42.4)	49 (57.6)
No	2170 (41.3)	3078 (58.7)

Differences between hypertension and no hypertension were analyzed by chi-squared test.

Statistical significance at

* p<0.05,

** p<0.01.

Table 4 displays the results of multivariate logistic regression analysis of the prevalence of hypertension by smoking, exposure to SHS, and drinking status after adjusting for age, sex, ethnicity, education level, annual household income, family history of hypertension, and obesity. Smokers (AOR=1.31; 95% CI: 1.13–1.51), individuals exposed to SHS (AOR=1.24; 95% CI: 1.11–1.43), drinkers (AOR=1.31; 95% CI: 1.15–1.50), and concurrent smokers and drinkers (AOR=1.45; 95% CI: 1.25–1.67) all had a higher probability of having hypertension (p<0.01). Additionally, concurrent smoking and drinking had the strongest association with the prevalence of hypertension (AOR=1.45; 95% CI: 1.25–1.67; p<0.01). Although we tested for possible interactions, there were no significant interactions between smoking or drinking and hypertension and sex.

**Table 4:** Logistic regression for prevalence of hypertension by smoking, secondhand smoke exposure (SHS), and drinking status among adults aged ≥35 years from a 2020–2021 cross-sectional study in rural Yunnan Province, China (N=7572)

*Variable*	*Hypertension (Ref: no)*	
	*AOR*	*95% CI*
Smokers (Ref: no)	1.31**	1.13–1.51
SHS exposure (Ref: no)	1.24**	1.11–1.43
Drinkers (Ref: no)	1.31**	1.15–1.50
Concurrent smoking and drinking (Ref: no)	1.45**	1.25–1.67

Adjusted for age, sex, ethnicity, education level, annual household income level, family history of hypertension, and obesity.

Statistical significance at

* p<0.05,

** p<0.01.

## DISCUSSION

The findings reveal that there are significant socioeconomic differences in the prevalence of tobacco exposure and drinking in rural southwest China. Further, it demonstrates smokers, individuals exposed to SHS, drinkers, and concurrent smokers and drinkers had a higher probability of having hypertension.

In the present study, while males smoked and drank more frequently than females, the prevalence of SHS exposure was higher in females than in males. These findings are in line with previous studies^22^. Furthermore, the prevalence of smoking and drinking among males in our study was higher than the prevalence rate observed in urban Chinese populations as well as that in other Asian countries^5,22,23^. Thus, effective smoking prevention and cessation measures should, in particular, target males to reduce male hypertension as well as prevent female SHS exposure. In addition, measures should be taken to attempt to reduce the intake of alcohol, especially among men.

Our study indicates ethnic disparities in the prevalence of SHS exposure and alcohol drinking; ethnic minorities had a higher prevalence of SHS exposure and alcohol drinking than Han participants. Previous research also demonstrated that ethnic minorities consume alcohol at higher levels than Han individuals^24^. This may result from genetic or cultural differences that lead to differing drinking behavior across ethnic minority groups^25^. The higher prevalence of SHS exposure among ethnic minorities suggests it is essential to strengthen awareness of tobacco hazards to reduce SHS, particularly in ethnic minority communities in rural China.

While several studies have shown that lower levels of education are associated with higher risks of smoking and environmental tobacco exposure^26^, our study showed that education level was positively associated with the prevalence of smoking, and there was no association between education level and SHS exposure. The causes of this inconsistent effect of education on smoking behaviors remain unclear, and further investigation is needed to examine the exact nature of the association between education and smoking behaviors in rural China. However, our results are consistent with prior studies indicating a positive effect of education on drinking behaviors, with people with a higher level of education more likely to drink frequently^27^. People with a higher level of education may be more likely to have higher socioeconomic status and thus stronger purchasing power for and higher consumption of alcohol^28^. Our results highlight the importance of targeting individuals with a higher level of education in efforts to reduce alcohol consumption.

Our study showed no association between individual income and smoking or drinking. These findings on smoking are in line with previous Chinese research^29^ but differ from findings in low- and middle-income countries where the prevalence of smoking was high in poor areas^30,31^. However, the relationship between income and drinking continues to be debated worldwide, with studies in high- and middle-income countries finding that people with high income were more likely to drink alcohol^32,33^ and in low-income countries were more likely to drink alcohol than low-income people^34^. More research is needed to fully uncover the nature of the relationship between income, drinking, and smoking.

The prevalence of hypertension in this study was 41.6%, a rate that exceeds that observed in other regions of China as well as other Asian countries^35,36^. Our findings, therefore, indicate that it is crucial to implement effective hypertension prevention and management measures in rural China. Furthermore, the findings indicate that smokers, individuals exposed to SHS, drinkers, and concurrent smokers and drinkers had a higher probability of having hypertension. This finding is consistent with previous research^16,17^. Our study in this way suggests that comprehensive blood pressure control measures should take into account health education about smoking and alcohol use. In addition, we observed an increase in the strength of the link between concurrent smokers and drinkers with hypertension, compared with smoking or drinking alone. Concurrent smoking and drinking can work together to raise blood pressure and significantly increase future hypertension risk^14^. Thus, our findings suggest reducing tobacco and alcohol use concurrently may improve health outcomes by lowering the risk of hypertension.

### Limitations

The study findings are limited in three ways. First, the self-reported prevalence of tobacco exposure and drinking is subject to recall bias, which may lead to underestimating the true prevalence of tobacco exposure and drinking in the study population. Second, the cross-sectional design of this study limits the ability to determine causal relationships. Third, the findings were based on a random sampling of three counties, limiting their generalizability.

## CONCLUSIONS

Socioeconomic factors play an important role in influencing the prevalence of smoking, exposure to SHS, and drinking in rural southwest China. Future hypertension interventions should focus on smokers, individuals exposed to SHS, drinkers, and, in particular, concurrent smokers and drinkers.

## Data Availability

The data supporting this research are available from the authors on reasonable request.

## References

[1] Wen H, Xie C, Wang F, Wu Y, Yu C. Trends in disease burden attributable to tobacco in China, 1990-2017: findings from the Global Burden of Disease Study 2017. Frontiers in public health. 2020;8:237. doi:10.3389/fpubh.2020.00237 32766191 PMC7381278

[2] Dai X, Gakidou E, Lopez AD. Evolution of the global smoking epidemic over the past half century: strengthening the evidence base for policy action. *Tob Control*. 2022;31(2):129-137. doi:10.1136/tobaccocontrol-2021-056535 35241576

[3] Zhou M, Wang H, Zeng X, et al. Mortality, morbidity, and risk factors in China and its provinces, 1990-2017: a systematic analysis for the Global Burden of Disease Study 2017. Lancet. 2019;394(10204):1145-1158. doi:10.1016/S0140-6736(19)30427-1 31248666 PMC6891889

[4] Huang YY, Di XB, Nan Y, et al. *Zhonghua Liu Xing Bing Xue Za Zhi*. 2022;43(6):824-829. doi:10.3760/cma.j.cn112338-20211130-00930 35725336

[5] Zhang M, Yang L, Wang L, et al. Trends in smoking prevalence in urban and rural China, 2007 to 2018: findings from 5 consecutive nationally representative cross-sectional surveys. PLoS Med. 2022;19(8):e1004064. doi:10.1371/journal.pmed.1004064 36006870 PMC9409540

[6] Chen Z, Peto R, Zhou M, et al. Contrasting male and female trends in tobacco-attributed mortality in China: evidence from successive nationwide prospective cohort studies. *Lancet*. 2015;386(10002):1447-1456. doi:10.1016/S0140-6736(15)00340-2 26466050 PMC4691901

[7] Alcohol use and burden for 195 countries and territories, 1990-2016: a systematic analysis for the Global Burden of Disease Study 2016. Lancet (London, England). 2018;392(10152):1015-1035. doi:10.1016/S2215-0366(18)30337-7 30146330 PMC6148333

[8] Shield K, Manthey J, Rylett M, et al. National, regional, and global burdens of disease from 2000 to 2016 attributable to alcohol use: a comparative risk assessment study. Lancet Public Health. 2020;5(1):e51-e61. doi:10.1016/S2468-2667(19)30231-2 31910980

[9] Manthey J, Shield KD, Rylett M, Hasan OSM, Probst C, Rehm J. Global alcohol exposure between 1990 and 2017 and forecasts until 2030: a modelling study. *Lancet*. 2019;393(10190):2493-2502. doi:10.1016/S0140-6736(18)32744-2 31076174

[10] Xu XL, Zhao LY, Fang HY, et al. Prevalence of drinking among residents aged 15 and above in China from 2010 to 2012. Journal of Hygiene Research. 2016;45(04): 534-537. doi:10.19813/j.cnki.weishengyanjiu.2016.04.005 29903318

[11] Pu W, Zhao LY, Fang HY, et al. Current status of alcohol consumption among adults aged 18 and above in China Chinese Food and Nutrition. 2021;27(10):15-19. doi:10.19870/j.cnki.11-3716/ts.2021.10.009

[12] Im PK, Wright N, Yang L, et al. Alcohol consumption and risks of more than 200 diseases in Chinese men. *Nat Med*. 2023;29(6):1476-1486. doi:10.1038/s41591-023-02383-8 37291211 PMC10287564

[13] NCD Risk Factor Collaboration (NCD-RisC). Worldwide trends in blood pressure from 1975 to 2015: a pooled analysis of 1479 population-based measurement studies with 19·1 million participants. Lancet. 2017;389(10064):37-55. doi:10.1016/S0140-6736(16)31919-5 27863813 PMC5220163

[14] Vallée A. Associations between smoking and alcohol consumption with blood pressure in a middle-aged population. *Tob Induc Dis*. 2023;21:61. doi:10.18332/tid/162440 37215190 PMC10193384

[15] Zhu B, Zhou J, Chen Y, et al. Incidence rate, risk factors and behaviour changes for alcohol drinking: findings from a community-based cohort study in Southwest China. *BMJ Open*. 2022;12(9):e060914. doi:10.1136/bmjopen-2022-060914 PMC9472170 36100302

[16] Gao N, Liu T, Wang Y, et al. Assessing the association between smoking and hypertension: smoking status, type of tobacco products, and interaction with alcohol consumption. *Front Cardiovasc Med*. 2023;10:1027988. doi:10.3389/fcvm.2023.1027988 36844742 PMC9947503

[17] Akpa OM, Okekunle AP, Asowata JO, Adedokun B. Passive smoking exposure and the risk of hypertension among non-smoking adults: the 2015-2016 NHANES data. *Clin Hypertens*. 2021;27(1):1. doi:10.1186/s40885-020-00159-7 33384019 PMC7775627

[18] Fan L, Liu L, Zhao Y, Mo Y, Li J, Cai L. Trends in the prevalence and economic burden of hypertension and its socioeconomic disparities in rural southwestern China: two repeated cross-sectional studies. *BMJ Open*. 2023;13(11):e076694. doi:10.1136/bmjopen-2023-076694 PMC10660421 37977876

[19] Cai L, Li X, Cui W, You D, Golden AR. Trends in diabetes and pre-diabetes prevalence and diabetes awareness, treatment and control across socioeconomic gradients in rural southwest China. *J Public Health (Oxf)*. 2018;40(2):375-380. doi:10.1093/pubmed/fdx097 28977385

[20] World Health Organization. WHO STEPS surveillance manual: the WHO STEPwise approach to chronic disease risk factor surveillance / noncommunicable diseases and mental health. WHO; 2005. Accessed May 19, 2024. https://iris.who.int/handle/10665/43376

[21] Perloff D, Grim C, Flack J, et al. Human blood pressure determination by sphygmomanometry. *Circulation*. 1993;88(5 Pt 1):2460-2470. doi:10.1161/01.cir.88.5.2460 8222141

[22] Suwanno J, Phonphet C, Mayurapak C, Ninla-Aesong P, Thiamwong L. Sex-based differences in risk of cardiovascular disease development and cardiovascular risk factors among individuals with hypertension: a cross-sectional study from primary care facilities. *J Vasc Nurs*. 2023;41(2):62-71. doi:10.1016/j.jvn.2023.04.002 37356872

[23] Li YR, Wang J, Zhao LY, et al. *Zhonghua Liu Xing Bing Xue Za Zhi*. 2018;39(7):898-903. doi:10.3760/cma.j.issn.0254-6450.2018.07.007 30060301

[24] Wang XM, Wu C, Golden AR, Le C. Ethnic disparities in prevalence and patterns of smoking and nicotine dependence in rural southwest China: a cross-sectional study. *BMJ Open*. 2019;9(9):e028770. doi:10.1136/bmjopen-2018-028770 PMC6756462 31542742

[25] Sue S. Epilogue for the special issue on sociocultural factors and mechanisms in alcohol use: epidemiology, prevention, and intervention among ethnic minority groups: lessons learned. *Am J Orthopsychiatry*. 2019;89(5):624-626. doi:10.1037/ort0000423 31436468

[26] Andersen AJ, Hecker I, Wallez S, et al. Are we equally at risk of changing smoking behavior during a public health crisis? Impact of educational level on smoking from the TEMPO cohort. *BMC Public Health*. 2023;23(1):1016. doi:10.1186/s12889-023-15799-1 37254131 PMC10227809

[27] Zhou T, Sun D, Li X, Ma H, Heianza Y, Qi L. Educational attainment and drinking behaviors: Mendelian randomization study in UK Biobank. *Mol Psychiatry*. 2021;26(8):4355-4366. doi:10.1038/s41380-019-0596-9 31768000 PMC7246132

[28] Lui CK, Kerr WC, Mulia N, Ye Y. Educational differences in alcohol consumption and heavy drinking: an age-period-cohort perspective. *Drug Alcohol Depend*. 2018;186:36-43. doi:10.1016/j.drugalcdep.2017.12.046 29544120 PMC6003414

[29] Cai L, Wang XM, Fan LM, Cui WL, Golden AR. Socioeconomic disparities in prevalence and behaviors of smoking in rural Southwest China. *BMC Public Health*. 2019;19(1):1117. doi:10.1186/s12889-019-7455-0 31412820 PMC6694669

[30] Xavier MO, Del-Ponte B, Santos IS. Epidemiology of smoking in the rural area of a medium-sized city in Southern Brazil. *Rev Saude Publica*. 2018;52(suppl 1):10s. doi:10.11606/S1518-8787.2018052000269 30234882 PMC6255251

[31] Paul B, Jean Simon D, Kondo Tokpovi VC, Kiragu A, Balthazard-Accou K, Emmanuel E. Tobacco use in Haiti: findings from demographic and health survey. *BMC Public Health*. 2023;23(1):2504. doi:10.1186/s12889-023-17409-6 38097954 PMC10720190

[32] Ormond G, Murphy R. The effect of alcohol consumption on household income in Ireland. *Alcohol*. 2016;56:39-49. doi:10.1016/j.alcohol.2016.10.003 27814793

[33] Kumar K, Kumar S, Singh AK. Prevalence and socio-demographic correlates of alcohol consumption: survey findings from five states in India. *Drug Alcohol Depend*. 2018;185:381-390. doi:10.1016/j.drugalcdep.2017.12.024 29544190

[34] Takahashi R, Wilunda C, Magutah K, Mwaura-Tenambergen W, Wilunda B, Perngparn U. Correlates of alcohol consumption in rural western Kenya: a cross-sectional study. *BMC Psychiatry*. 2017;17(1):175. doi:10.1186/s12888-017-1344-9 28486959 PMC5424353

[35] Fan G, Jiang Z, Li J, Shi L, Gui C, Huang R. Prevalence, awareness, treatment and control of hypertension in Guangxi Zhuang Autonomous Region. *Sci Rep*. 2022;12(1):900. doi:10.1038/s41598-021-04735-1 35042905 PMC8766488

[36] Chowdhury MAB, Islam M, Rahman J, Uddin MT, Haque MR, Uddin MJ. Changes in prevalence and risk factors of hypertension among adults in Bangladesh: an analysis of two waves of nationally representative surveys. *PLoS One*. 2021;16(12):e0259507. doi:10.1371/journal.pone.0259507 34855768 PMC8638884

